# Prediction of an oxygen extraction fraction map by convolutional neural network: validation of input data among MR and PET images

**DOI:** 10.1007/s11548-021-02356-7

**Published:** 2021-04-05

**Authors:** Keisuke Matsubara, Masanobu Ibaraki, Yuki Shinohara, Noriyuki Takahashi, Hideto Toyoshima, Toshibumi Kinoshita

**Affiliations:** 1 Department of Radiology and Nuclear Medicine, Research Institute for Brain and Blood Vessels, Akita Cerebrospinal and Cardiovascular Center, 6-10 Senshu-Kubota-machi, Akita, 010-0874 Japan; 2 Preparing Section for New Faculty of Medical Science, Fukushima Medical University, Fukushima, Japan

**Keywords:** Deep learning, Positron emission tomography, Oxygen-15, Oxygen extraction fraction, Stroke

## Abstract

**Purpose:**

Oxygen extraction fraction (OEF) is a biomarker for the viability of brain tissue in ischemic stroke. However, acquisition of the OEF map using positron emission tomography (PET) with oxygen-15 gas is uncomfortable for patients because of the long fixation time, invasive arterial sampling, and radiation exposure. We aimed to predict the OEF map from magnetic resonance (MR) and PET images using a deep convolutional neural network (CNN) and to demonstrate which PET and MR images are optimal as inputs for the prediction of OEF maps.

**Methods:**

Cerebral blood flow at rest (CBF) and during stress (sCBF), cerebral blood volume (CBV) maps acquired from oxygen-15 PET, and routine MR images (T1-, T2-, and T2*-weighted images) for 113 patients with steno-occlusive disease were learned with U-Net. MR and PET images acquired from the other 25 patients were used as test data. We compared the predicted OEF maps and intraclass correlation (ICC) with the real OEF values among combinations of MRI, CBF, CBV, and sCBF.

**Results:**

Among the combinations of input images, OEF maps predicted by the model learned with MRI, CBF, CBV, and sCBF maps were the most similar to the real OEF maps (ICC: 0.597 ± 0.082). However, the contrast of predicted OEF maps was lower than that of real OEF maps.

**Conclusion:**

These results suggest that the deep CNN learned useful features from CBF, sCBF, CBV, and MR images and predict qualitatively realistic OEF maps. These findings suggest that the deep CNN model can shorten the fixation time for ^15^O PET by skipping ^15^O_2_ scans. Further training with a larger data set is required to predict accurate OEF maps quantitatively.

**Supplementary Information:**

The online version contains supplementary material available at 10.1007/s11548-021-02356-7.

## Introduction

Oxygen extraction fraction (OEF) is a biomarker of the viability of brain tissue in ischemic stroke [1–4]. Positron emission tomography (PET) with oxygen-15 gases (^15^O PET) is the gold standard method for quantifying OEF maps [5, 6]. Calculating the OEF map requires PET scans for cerebral blood flow (CBF) with C^15^O_2_ or H_2_^15^O and cerebral blood volume (CBV) with C^15^O, as well as a ^15^O_2_ scan. Arterial blood sampling is also required to quantify the OEF, CBF, and CBV in ^15^O PET. The long fixation time of the ^15^O PET scans, which consists of preparing the arterial blood sampling and three PET scans (1–2 h), places a burden on patients. These issues prevent the widespread use of ^15^O PET in clinical settings. Kudomi and colleagues proposed a method of shortening the total fixation time by continuous inhalation of C^15^O_2_ and ^15^O_2_ gases [7]. However, the continuous inhalation protocol has not been widely used. Various methods to acquire OEF maps only with magnetic resonance (MR) imaging data [8–12] were proposed previously. These methods have not also been widely used in clinical due to the need for special calculation and sequences.

Deep learning, which is a type of machine learning with a neural network consisting of numerous layers [13, 14], has been recently and widely used in the computer vision area. Deep-learning techniques, such as a convolutional neural network (CNN) and generative adversarial network, have been applied for image synthesis and transformation between different images as follows: denoizing/superresolution [15, 16], synthesis of computed tomography (CT) images from MR images [17–19], motion correction [20], missing data recovery [21], and image reconstruction [22–24]. To map ischemic stroke, prediction of CBF [25] and cerebrovascular reserve [26] maps using the CNN learned with arterial spin labeling (ASL) maps and structural MR images have been proposed.

We hypothesized that the deep CNN could predict OEF maps without the ^15^O_2_ scan from the other PET and MR images. To verify this hypothesis, we performed learning of structural MR images, CBV maps, and CBF maps at rest and under stress with acetazolamide as inputs and OEF maps as a target with U-shaped CNN with skip connections (U-Net) [27]. To demonstrate which MRI, CBF, and CBV are optimal for predicting the OEF maps, we compared the models learned with various combinations of MR images, CBF, and CBV maps as inputs. Finally, we performed the test using the model learned with the best combination.

## Materials and methods

### Data

We retrospectively analyzed data from patients with unilateral cerebrovascular steno-occlusive disease who underwent both MR and ^15^O PET scans as part of their routine preoperative examination between 2011 and 2018 (*n* = 138; age: 65.9 ± 10.3 [range: 27–85] years; female/male = 29/109). This study was performed in accordance with the Ethical Guideline for Clinical Research, issued by the Ministry of Health, Labor and Welfare of the Japanese government (2008), and was approved by the Ethics Committee of Akita Cerebrospinal and Cardiovascular Center (no. 20-01).

We regarded the most recent data for 25 subjects (age: 63.8 ± 10.5 [37–80] years; female/male = 4/21) from the data set as test data, and the remaining data for 113 subjects (age: 66.3 ± 10.3 [27–85] years; female/male = 25/88) as training/validation data.

### Scan procedures and image processing

PET data were acquired using an SET-3000GCT/M scanner (Shimadzu Corp., Kyoto, Japan) dedicated to the three-dimensional (3D) acquisition mode [28]. The details of ^15^O PET scans have been described elsewhere [29]. Motion correction for the ^15^O PET scans was performed using a previously described software-based method [30]. The OEF maps were estimated using the autoradiographic method [6] based on images acquired through inhaled ^15^O_2_ and CBF estimated from H_2_^15^O PET images [31]. Blood volume was subtracted from the OEF maps by CBV estimated from C^15^O PET images. The stressed CBF maps were estimated using data acquired with H_2_^15^O and acetazolamide, as described in previous reports [30, 32].

MR scans to acquire T1-, T2-, and T2*-weighted images were performed with a 3T MR scanner (Verio Dot, Siemens Healthcare, Germany). T1-weighted images were acquired using a turbo spin echo (TSE) sequence with dark fluid technology. The parameters of the T1-weighted image sequences were as follows: repetition time (TR): 2000 ms; echo time (TE): 9.5 ms; inversion time (TI): 858 ms; flip angle: 120°; slice thickness: 5 mm; gap: 1 mm; matrix size: 320 × 320. T2-weighted images were acquired using a TSE sequence with the following parameters: TR: 4000 ms; TE: 93 ms; flip angle: 145°; slice thickness: 5 mm; gap: 1 mm; matrix size: 512 × 512. T2*-weighted images were acquired using a gradient echo sequence with the following parameters: TR: 680 ms; TE: 16 ms; flip angle: 20°; slice thickness: 5 mm; gap: 1 mm; matrix size: 384 × 384.

The T2- and T2*-weighted images and CBF map at rest were registered to the T1-weighted images. The transformation matrix was applied to the realignment of OEF and CBV maps to the T1-weighted images. Then, all images were realigned to spaces for the T1-weighted images. These registration processes were performed using the FreeSurfer software package (https://surfer.nmr.mgh.harvard.edu/). Each slice for the realigned images was down-sampled to 256 × 256 for the input and target data for the deep CNN model. All images were standardized by the average for an individual image.

For extracting brain mask and the region-of-interest analysis described below, spatial normalization of the T1-weighted images was performed using the unified segmentation algorithm [33]. The deformation estimated for the T1-weighted images was applied for the other realigned MR images and CBF, CBV, and OEF maps. These spatial normalization processes were performed using the SPM12 software package (https://www.fil.ion.ucl.ac.uk/spm/).

Flowchart for the image pre-processing is shown in Fig. S1 on Supplementary Materials.

### Training

**Fig. 1:**
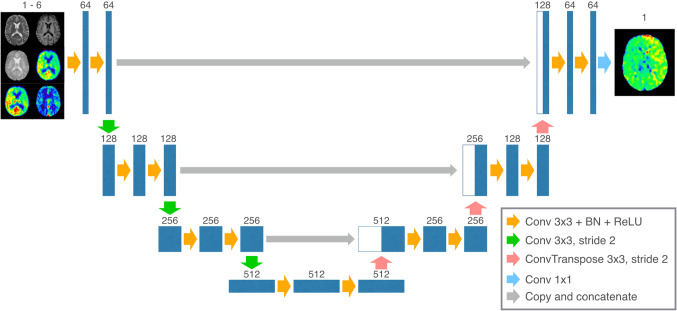
The U-Net model used in this study. The numbers on each layer indicate the number of channels

Figure 1 illustrates the U-Net used in this study. The U-Net was trained with the training data set for the 113 subjects. Briefly, the U-Net contains an encoder part to compress data for extracting robust image features and a decoder part to restore a desirable image from the extracted features. The decoder part has a mirrored structure of the encoder part. Each level of the encoder and decoder parts contains two convolutional layer blocks. Each block contains a convolutional layer, a batch normalization layer to avoid internal covariance shifts [34], and an activation layer with a rectified linear unit [35]. Up-sampling on the decoder part was implemented with a transposed convolutional layer with stride by 2. Down-sampling on the encoder was implemented with a convolutional layer with stride by 2, instead of a pooling layer, due to improving the ability of expression for the network [36]. The level of down- and up-sampling was set to 3 empirically. To avoid losses of spatial information, the skip connections were added on each level. Finally, the output images were recovered from the final image features using a convolutional layer with a 1 × 1 kernel.

Weights on the network were optimized by minimizing the mean squared error between the real and predicted OEF maps. The optimization of the weights was performed using the Adam algorithm [37]. We used the default values of hyperparameters for Adam in this study, except for β_1_, which was set to 0.5 empirically. The update of the weights was implemented by a batch, including eight image data sets, and iterated with 100 epochs. The initial learning rate was set to 0.001. The learning rate was linearly decayed from the 50th epoch to the end of the learning. We performed data augmentation of the training data with rotation and a horizontal flip. The training processes were implemented using the PyTorch library (https://pytorch.org) [38].

### Validation for combination of input images

To validate which combination among structural MR images, rest CBF, stressed CBF, and CBV maps were optimal for predicting the OEF maps, we performed the training using various combinations of input images, as presented in Table 1. To simplify the validation, we regarded the structural MR image data set including T1-, T2-, and T2*-weighted images as one image data type with three channels, which was termed “MRI.” Stressed CBF was denoted as “sCBF” hereafter. The models were validated with five-fold cross-validation. Briefly, we split the 113 training/validation data into five data subsets, regarded four and one subsets as training and validation data, respectively, and then repeated training and evaluation of the trained model five times such that all subsets had been validated.

**Table 1:** Combination of input images to validate. sCBF indicates CBF on stressed by acetazolamide

	Combinations
Single-image types	MRI; CBF; sCBF; CBV
Two-image types	MRI + CBF; MRI + sCBF; MRI + CBV; CBF + sCBF; CBF + CBV; CBV + sCBF
Three-image types	MRI + CBF + sCBF; MRI + CBF + CBV; MRI + CBV + sCBF; CBF + CBV + sCBF
Full model	MRI + CBF + CBV + sCBF

We calculated the intraclass correlation for agreement between the predicted and real OEF values on brain voxels (ICC (2, 1)) as an index for performance to predict the OEF map. The real and predicted OEF maps were down-sampled by 4 for rapid calculation of the ICC. We compared the ICC among the models learned using the combination of input images. Individual brain masks were calculated using spatial normalization to the Montreal Neurological Institute (MNI) template and the inverse transformation to the individual brain. We performed the Dunnett’s test to test the differences from the model with the best ICC. We also calculated the effect sizes of the differences in ICC.

A four-way repeated-measures analysis of variance (ANOVA) was performed to demonstrate which images contributed to the prediction performance. Binary variables, as to whether each image (MRI, CBF, CBV, and sCBF) was used as an input image, were regarded as independent variables. For each independent variable, we calculated the partial eta-squared (*η*_p_^2^) as an effect size to contribute to the ICC values. ICC values were regarded as dependent variables. The ICC was calculated with the Pingouin library (https://pingouin-stats.org/index.html) [39]. The ANOVA was performed with the R programming language (https://www.r-project.org/).

To extract cortical OEF values, volume-of-interests (VOI) template, based on labeled data provided by Neuromorphometrics, Inc. (http://Neuromorphometrics.com) under academic subscription, was applied on the spatially normalized OEF maps. The template VOIs were masked with a gray matter mask, determined with thresholding of the tissue probability map on the MNI template by 0.5. OEF values for the cerebral cortex on each hemisphere were calculated from the average values on nine cortical regions were extracted. The nine cortical regions consisted of the frontal, parietal, occipital, temporal, central operculum, anterior cingulate, middle cingulate, posterior cingulate, and insula cortices. Ratio of OEF between ipsi- and contra-lateral VOIs for the cortical regions was calculated.

### Test

To assess the generalization performance of the trained model, the model with the best prediction performance in the validation was tested using the test data for the most recent 25 subjects. The model trained with all training data for 113 subjects was tested. The ICC for the predicted OEF values on the brain voxels was calculated in a similar manner to the validation.

## Results

### Validation for the combination of input images

**Fig. 2:**
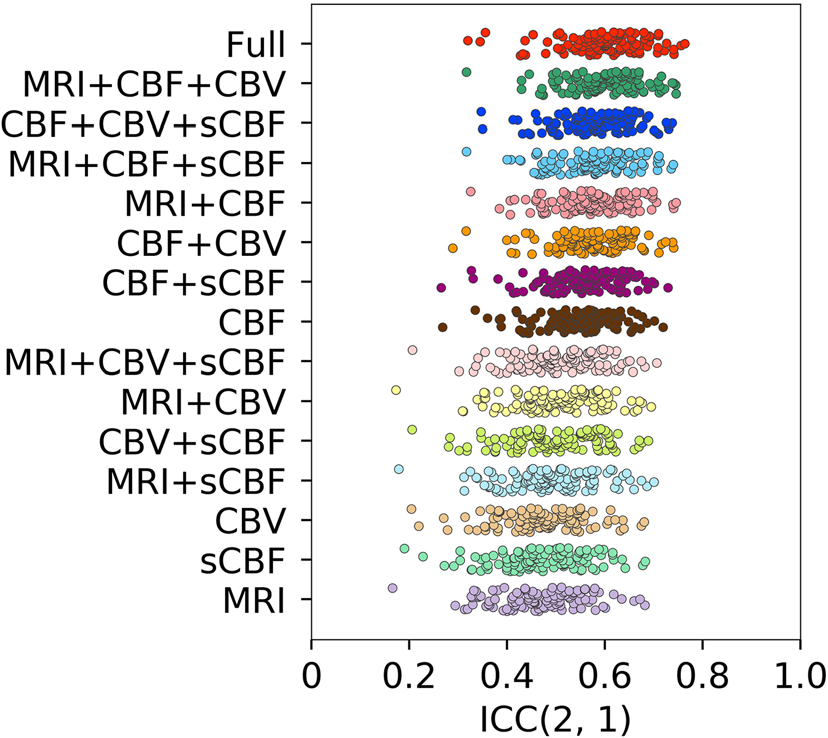
Plot for the individual ICC between the real and predicted OEF values on the brain voxels for the validation data sets. Note that each dot indicates an individual from the data pooled from the validation data sets

**Fig. 3:**
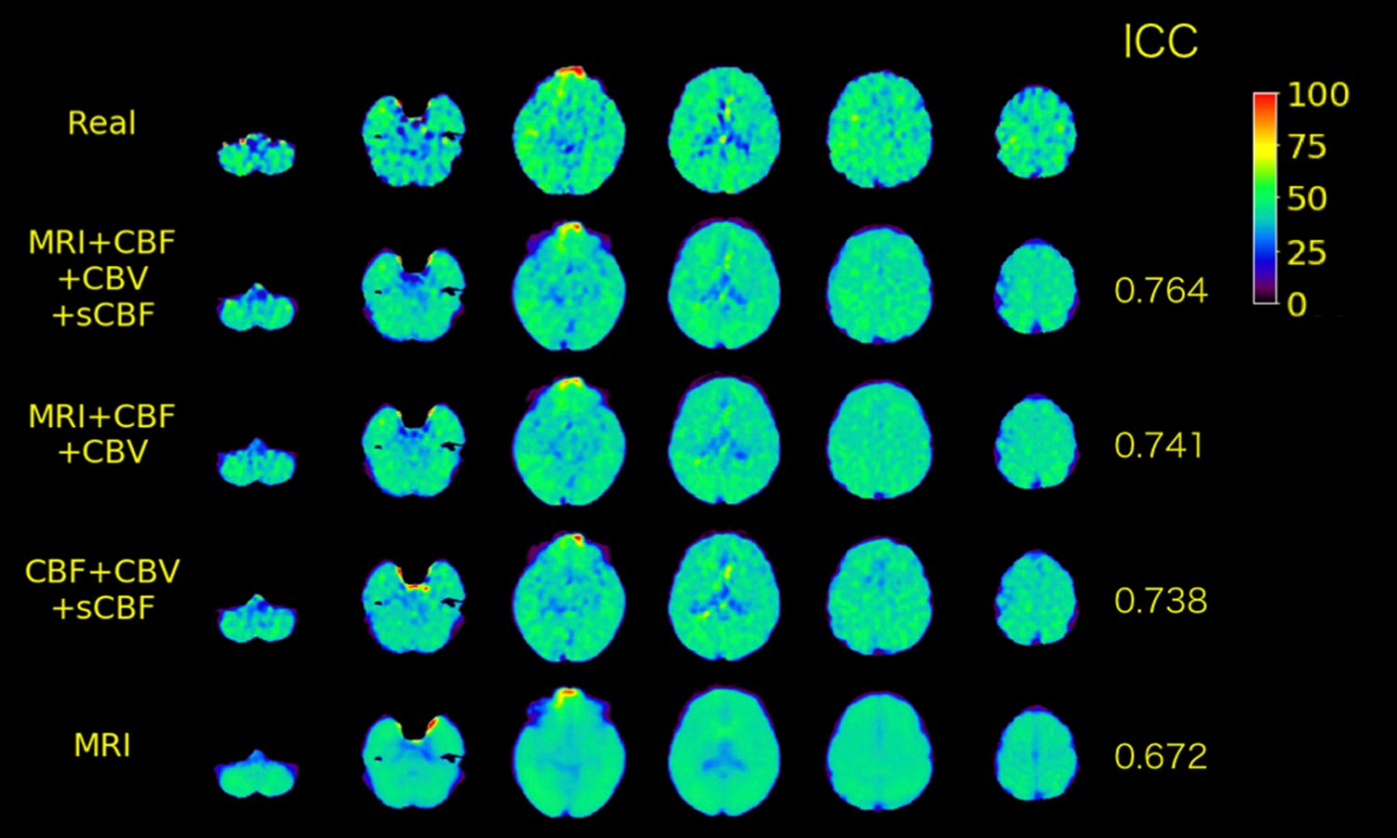
Real and predicted OEF maps for a case (77 years old, male, right internal carotid artery occlusion), with the highest ICC by the full model (MRI + CBF + CBV + sCBF) in the validation data set. The map on the top indicates the real OEF map. The maps in the three rows in the center indicate the OEF maps predicted by the model with the top-three mean ICC among the validation data set (full model; MRI + CBF + CBV; CBF + CBV + sCBF). The bottom map indicates the OEF maps predicted by the model with the worst mean ICC (MRI). ICC values for each model are also shown on the right

As presented in Fig. 2 and Table 2, the highest ICC (0.597 ± 0.082) to the real OEF values was observed with the model learned with MRI, CBF, CBV, and sCBF images (full model). The OEF maps predicted by the full model were similar to those of the real OEF maps, as illustrated in Fig. 3. The ICC value for the full model (0.597) indicates moderate agreement between the real and predicted OEF maps. No significant difference in ICC was observed among the models with the top-six mean ICC. In the case illustrated in Fig. 3, we did not observe marginal differences among the OEF maps predicted by the models with a top-three ICC. The ICC for the model other than top-six was significantly lower than the ICC for the full model. The large effect sizes (> 1.6) for the differences of ICC to the full model were observed with the model learned without resting CBF. The model learned only with MRI resulted in the worst mean ICC and the flat OEF maps, as illustrated in Fig. 3.

**Table 2:** ICC between predicted and real OEF values on brain voxels for the model learned with each combination of the input images

Combination	ICC	*t* value	*p* value	Effect size
MRI + CBF + CBV + sCBF [Full model]	0.597 ± 0.082 [0.320–0.764]			
MRI + CBF + CBV	0.596 ± 0.075 [0.317–0.746]	− 0.052	1.000	0.016
CBF + CBV + sCBF	0.577 ± 0.078 [0.347–0.738]	− 1.776	0.473	0.420
MRI + CBF + sCBF	0.574 ± 0.079 [0.317–0.740]	− 2.048	0.297	0.490
MRI + CBF	0.574 ± 0.080 [0.326–0.746]	− 2.086	0.275	0.533
CBF + CBV	0.572 ± 0.078 [0.289–0.741]	− 2.186	0.226	0.581
CBF + sCBF	0.553 ± 0.080 [0.266–0.729]	− 3.927	0.001	0.756
CBF	0.542 ± 0.080 [0.268–0.719]	− 4.902	< 0.001	0.885
MRI + CBV + sCBF	0.508 ± 0.087 [0.206–0.706]	− 7.978	< 0.001	1.603
MRI + CBV	0.501 ± 0.089 [0.173–0.695]	− 8.554	< 0.001	1.794
CBV + sCBF	0.496 ± 0.093 [0.206–0.689]	− 9.009	< 0.001	1.680
MRI + sCBF	0.490 ± 0.090 [0.179–0.701]	− 9.580	< 0.001	1.918
CBV	0.471 ± 0.087 [0.204–0.680]	− 11.262	< 0.001	2.003
sCBF	0.471 ± 0.091 [0.190–0.683]	− 11.301	< 0.001	1.880
MRI	0.468 ± 0.087 [0.166–0.682]	− 11.537	< 0.001	2.141

*t* values, *p* values, and effect size for the differences in ICC to the best model (MRI + CBF + CBV + sCBF) are also shown. The *t* and *p* values were calculated using the Dunnett’s test. Values in the “ICC” column indicate the mean ± standard deviation (minimum–maximum). Data in the table are sorted in descending order of mean ICC values

**Fig. 4:**
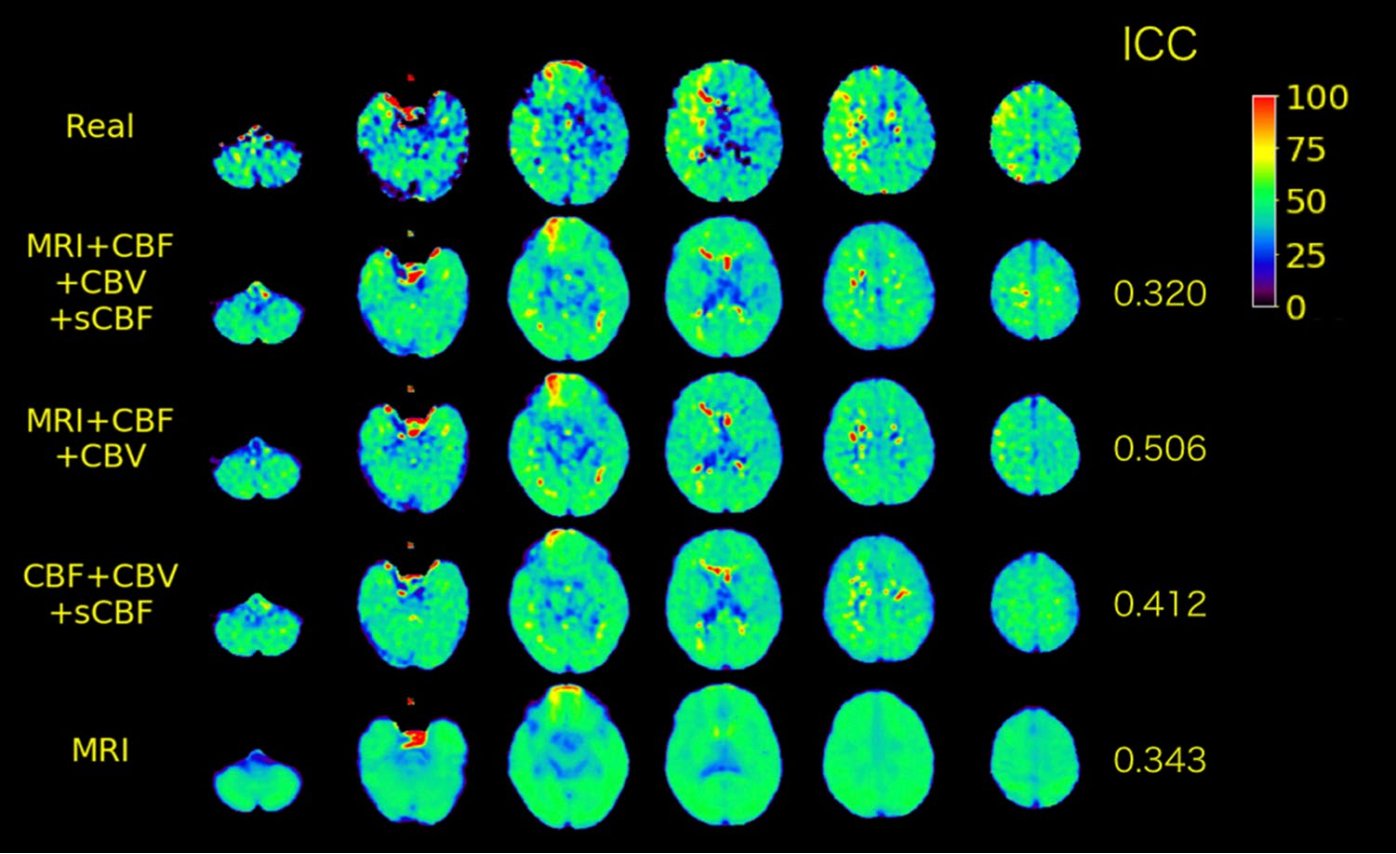
Real and predicted OEF maps for a case (71 years old, male, right internal carotid artery stenosis) with the lowest ICC by the full model (MRI + CBF + CBV + sCBF) among the validation data set. The legends are the same as those in Fig. 3

**Fig. 5:**
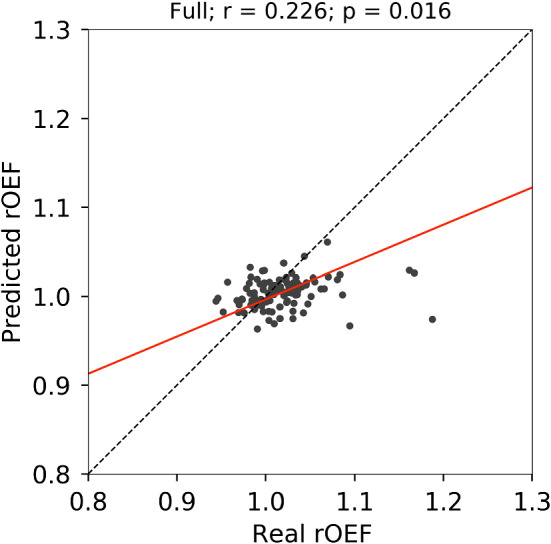
Scatter plot of rOEF values on cerebral cortex for the validation data between real and predicted with the full model. Red line indicates a regression line. Dashed line indicates perfect correspondence

In the case illustrated in Fig. 4, we observed a lower contrast on the predicted OEF maps, even with the full model, as compared with the real OEF maps. This case resulted in the lowest ICC with the full model among the validation data set and had high laterality of OEF. Similar trends were observed in other cases with high laterality of OEF, as shown in Fig S2 on Supplementary Materials. The lower predicted rOEF values than real ones were observed in the cases with the higher real rOEF, as shown in Figs. 5 and S3. In the cases with low real rOEF due to cerebral infarction, as illustrated in Fig. S4, the predicted rOEF values were higher than the real rOEF values.

Table 3 indicates that all binary variables for the input images had significant effects on ICC. We observed a much stronger effect of CBF on ICC (*η*_p_^2^ = 0.576) than that of the other variables. The effects of CBV (*η*_p_^2^ = 0.096) and MRI (*η*_p_^2^ = 0.065) on ICC were moderate. The effect of stressed CBF (*η*_p_^2^ = 0.021) was the weakest among the binary variables.

**Table 3:** Results for the four-way repeated-measures ANOVA with binary variables, based on whether each image was used as an input image

Variables	Degree of freedom	Sum of squares	Mean squares	F values	*p* values	*η*_p_^2^
is_CBF	1	3.175	3.175	2297.61	< 0.001	0.593
is_MRI	1	0.146	0.146	105.87	< 0.001	0.063
is_CBV	1	0.227	0.227	164.02	< 0.001	0.094
is_sCBF	1	0.048	0.048	34.94	< 0.001	0.022
Subjects	112	9.703	0.087			
Residuals	1578	2.180	0.001			

Effect sizes (*η*_p_^2^) indicating the effect of each binary variable are also shown. “is_XXX” means the binary variable based on whether image “XXX” was used as an input image

For the test below, we applied the full model because it had the best ICC and the most significant effects of all input images on ICC.

### Test

**Fig. 6:**
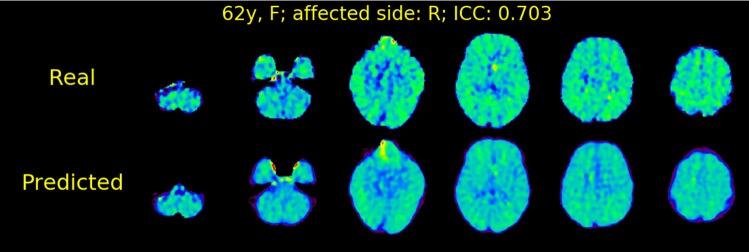
Real and predicted OEF maps for a case (62 years old, female, right internal carotid artery stenosis) with the highest ICC in the test data set

**Fig. 7:**
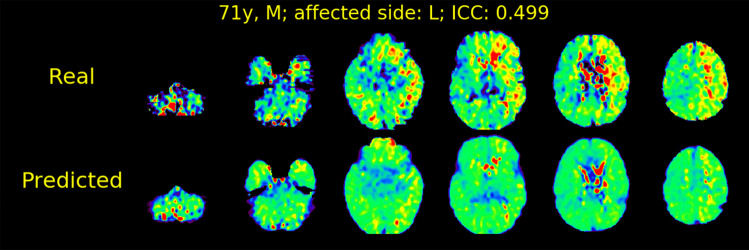
Real and predicted OEF maps for a representative case (71 years old, male, left internal carotid artery stenosis) with high laterality and relatively low ICC in the test data set

**Fig. 8:**
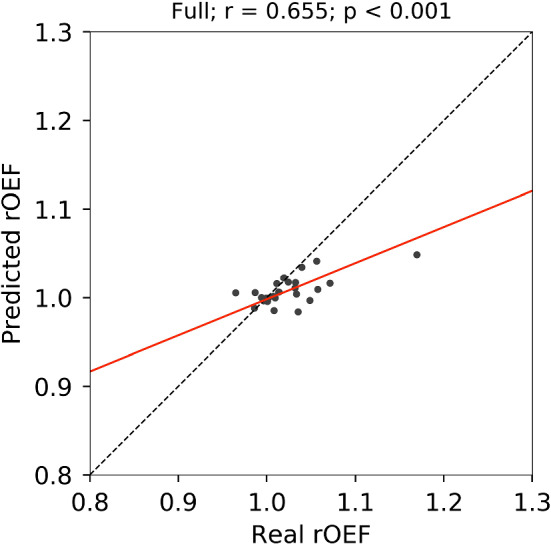
Scatter plot of rOEF values on cerebral cortex for the test data between real and predicted with the full model. The legends are the same as those in Fig. 5

The mean ICC value for the test data sets was 0.591 ± 0.081. We observed no significant differences in ICC between validation and test data sets (Welch’s *t* test: *t* = 0.342; *p* = 0.734; effect size = 0.075). As illustrated in Fig. 6, we observed similar textures to the real OEF map in the predicted OEF map for a case with the highest ICC (0.703). However, the predicted OEF values in this case were apparently lower on the whole brain than the real OEF values were. The underestimation of OEF values was observed in 12 cases in the test data sets, as illustrated in Fig. 6. In the case illustrated in Fig. 7, lower contrast on the predicted OEF maps than that on the real OEF maps was observed; this case had high laterality of OEF, similar to that observed in the validation data set illustrated in Fig. 4. Similar trends in predicted cortical rOEF values to the validation, underestimation in the case with high real rOEF, were observed in the test dataset, as illustrated in Fig. 8.

## Discussion

We attempted to predict OEF maps without using the ^15^O_2_ scan through machine learning of the other PET and MR images with deep CNN. The predicted OEF maps were similar to the real maps, and the moderate ICC values obtained by the model learned with MRI, CBF, CBV, and stressed CBF maps indicate that deep CNN trained with the PET and MR images can qualitatively predict OEF maps without the ^15^O_2_ scan. This finding suggests that by skipping the ^15^O_2_ scan, the trained deep CNN can shorten the fixation time for the ^15^O PET scans.

The best ICC for the full model and the significant effects of all binary variables for the input images suggest that all MRI, CBF, CBV, and stressed CBF maps contribute to the prediction of the OEF map. The very strong effect of the resting CBF on the prediction was as we expected, because the OEF maps as the target were calculated with the resting CBF maps using the autoradiographic method. The contribution of the CBV maps indicates that the deep CNN model learned an association between the dilation of blood vessels and oxygen supply to the brain tissue in stroke. The contribution of stressed CBF indicates that the deep CNN model can learn the relation between cerebrovascular reactivity and OEF. The cerebrovascular reactivity, measured by the difference between rest and stressed CBF, decreases in advance of the elevation of OEF in stroke. The contribution of the MRI indicates that the CNN model learned two pieces of information from the MRI: One is the anatomical information for the individual brain, and the other information is the information on changes in susceptibility with deoxygenation of hemoglobin. The elevation in OEF results in changes in intensities in vessels in T2*-weighted images [40]. These findings suggest that all MRI, CBF, CBV, and stressed CBF maps are useful as input images for the prediction of OEF maps using the deep CNN model. Therefore, for this study, we selected the full model trained with MRI, CBF, CBV, and sCBF maps for the test.

The moderate ICC in the test data set was the same as that in the validation data set; this suggests that the trained CNN model was successfully generalized, except for cases with high laterality of OEF. The trained model failed to predict the OEF maps with high laterality, and resulted in the underestimation of rOEF values in the validation and test data sets. The overestimation of rOEF was observed in the cases with the lower real rOEF than 1.0 due to cerebral infarction. These results reflect the lack of training data, as only a few cases with high laterality and low rOEF were included in the training data set in this study. To quantitatively predict accurate OEF maps, further training of a larger number of cases with high laterality of OEF and low rOEF is required.

Another limitation of this study is that because the features learned by the deep CNN model are too complicated for humans, we cannot understand what the model has learned. However, the similarity of the predicted OEF maps to the real maps and the moderate ICC suggests that the deep CNN model has learned useful features for the prediction of OEF maps from MRI, CBF, CBV, and sCBF maps. Further studies are required to interpret the model using techniques such as attention network [41, 42].

The findings in this study suggest that the trained deep CNN can shorten the fixation time for ^15^O PET scans by approximately 15 min by skipping ^15^O_2_ scans. The good prediction of OEF maps with deep CNN trained with MR and CBF images (“MRI + CBF” model) as similar to the full model imply that we can shorten ^15^O PET scans by 30 min, by skipping C^15^O scan as well as ^15^O_2_ scan. However, the scans with ^15^O-water or C^15^O_2_ gases, arterial blood sampling, and in-house cyclotron are still required even if C_15_O and ^15^O_2_ scans can be skipped. The contributions of CBF maps to the prediction of OEF maps also imply that CBF maps acquired by perfusion imaging with MR such as the ASL method, and single photon emission computed tomography can be used as alternative training data for the prediction of OEF maps. Further studies are required to demonstrate the validity of the maps acquired by methods other than ^15^O PET.

In conclusion, the results in this study suggest that the trained deep CNN model can qualitatively predict OEF maps. To predict OEF maps, the deep CNN model can learn useful features from MRI, rest CBF, CBV, and stressed CBF. These findings suggest that by skipping the ^15^O_2_ scan, the trained deep CNN model can shorten the fixation time for ^15^O PET. However, training with a larger data set is required for the prediction of quantitative OEF maps.

## Supplementary Information

Below is the link to the electronic supplementary material.

Supplementary file1 (DOCX 962 kb)

## Data Availability

Please contact the corresponding author for data requests.

## Code availability

Please contact the corresponding author for requests of custom codes used in this study.
