# Supplementary material for: Prediction of an oxygen extraction fraction map by convolutional neural network: validation of input data among MR and PET images
Source: Int J Comput Assist Radiol Surg. 2021 Apr 5;16(11):1865–74. doi: 10.1007/s11548-021-02356-7 (PMC8589760; doi:10.1007/s11548-021-02356-7)
Supplement: Supplementary file 1 — Supplementary file1 (DOCX 962 kb) [file 11548_2021_2356_MOESM1_ESM.docx]

**Supplementary Materials**


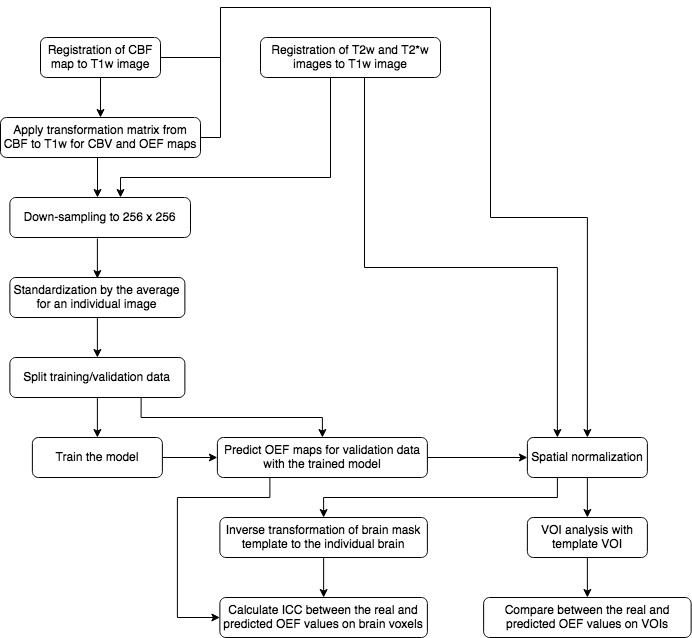


Fig. S1 Flowchart for image pre- and post-processing.


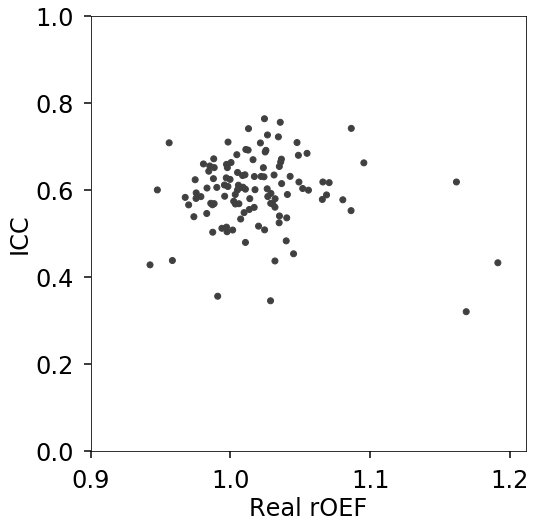


Fig. S2 Scatter plot between real OEF ratio (rOEF = ipsilateral OEF / contralateral OEF) and ICC between real and predicted OEF. ICC shown in the plot is for the full model. The real rOEF values were calculated from averaged values among the nine cortical regions on each hemisphere.


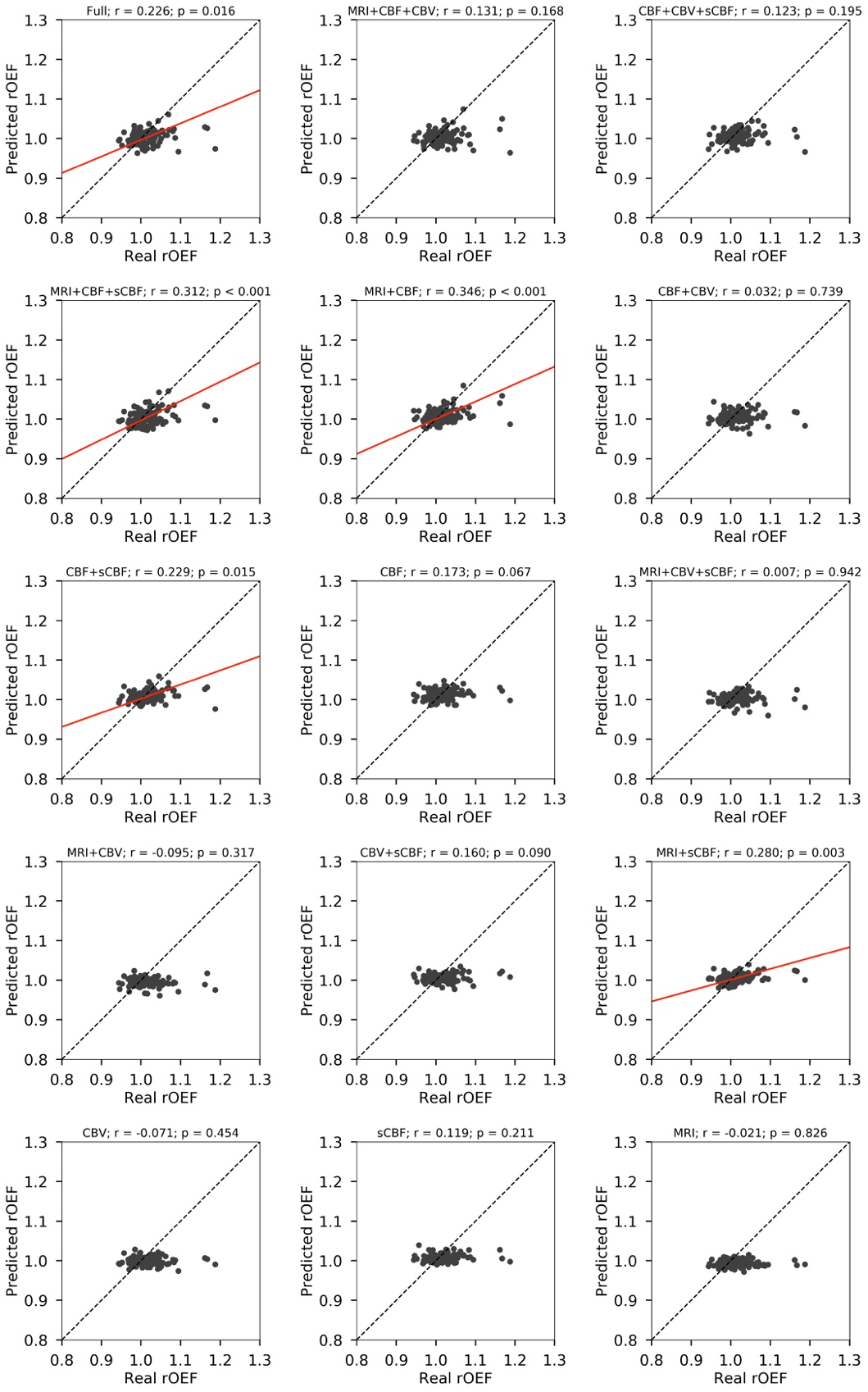


Fig. S3 Scatter plot of rOEF values on cerebral cortex for the validation data between real and predicted with each model. Note that the first plot indicates the results for the full model, and thus is same as Fig. 5. Red line indicates a regression line. Dashed line indicates perfect correspondence.


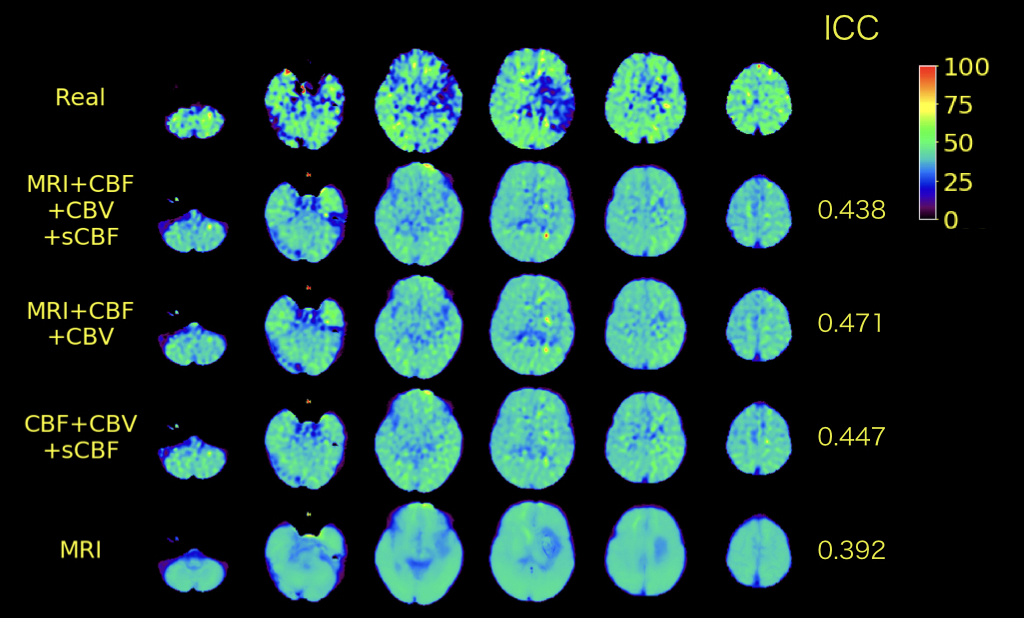


Fig. S4 Real and predicted OEF maps for a case (63 years old, male, left internal carotid artery occlusion), with lower real rOEF than 1.0 due to cerebral infarction in the validation data set. The map on the top indicates the real OEF map. The maps in the three rows in the center indicate the OEF maps predicted by the model with the top-three mean ICC among the validation data set (full model; MRI + CBF + CBV; CBF + CBV + sCBF). The bottom map indicates the OEF maps predicted by the model with the worst mean ICC (MRI). ICC values for each model are also shown on the right.
